# Supplementary material for: On the limits of 16S rRNA gene-based metagenome prediction and functional profiling
Source: Microb Genom. 2024 Feb 29;10(2):001203. doi: 10.1099/mgen.0.001203 (PMC10926695; doi:10.1099/mgen.0.001203)
Supplement: Supplementary material 1 [file mgen-10-1203-s001.pdf]

3.1 Correlation is not a suitable performance measure for metagenome prediction tools

Sun *et al.* [1] previously investigated the performance of metagenome prediction tools and observed that the comparably high Spearman correlation values are unaffected by label permutation. We could confirm these findings on four independent disease cohorts. The results showed that PICRUSt2, Tax4Fun2, and PanFP achieved Spearman correlation values ranging from 0.65 to 0.75 **(Figure S1),** which did not drop significantly after sample label permutation. MetGEM performed slightly worse than its competitors. Using rrnDB copy number normalization, PICRUSt2, Tax4Fun2, and MetGEM showed little improvement. Meanwhile, the performance of PanFP was raised to the level of the top-performing tool PICRUSt2. Since correlation analysis is not suited to robustly assess the performance of existing methods, differential abundance testing was used as an alternative measure in the main manuscript.

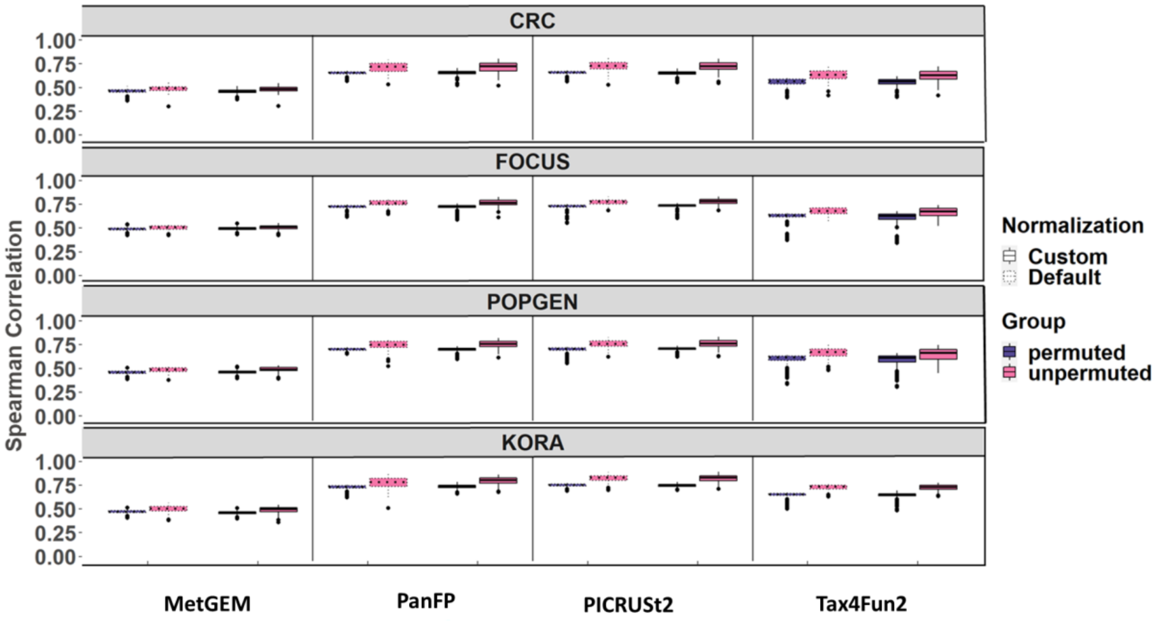

Figure S1: Spearman correlations between metagenome predictions and shotgun metagenome sequencing in unpermuted and permuted datasets. Validation of functional prediction tools comparing metagenome prediction performance against MGS as a gold standard. Spearman correlations of gene composition estimated from metagenome sequencing and predicted with PICRUSt2, Tax4Fun, PanFP, and MetGEM with default and customized normalization in unpermuted (blue) and permuted data (red) in all datasets. In each of the 100 permutations, every gene's abundance was permuted across samples independently.

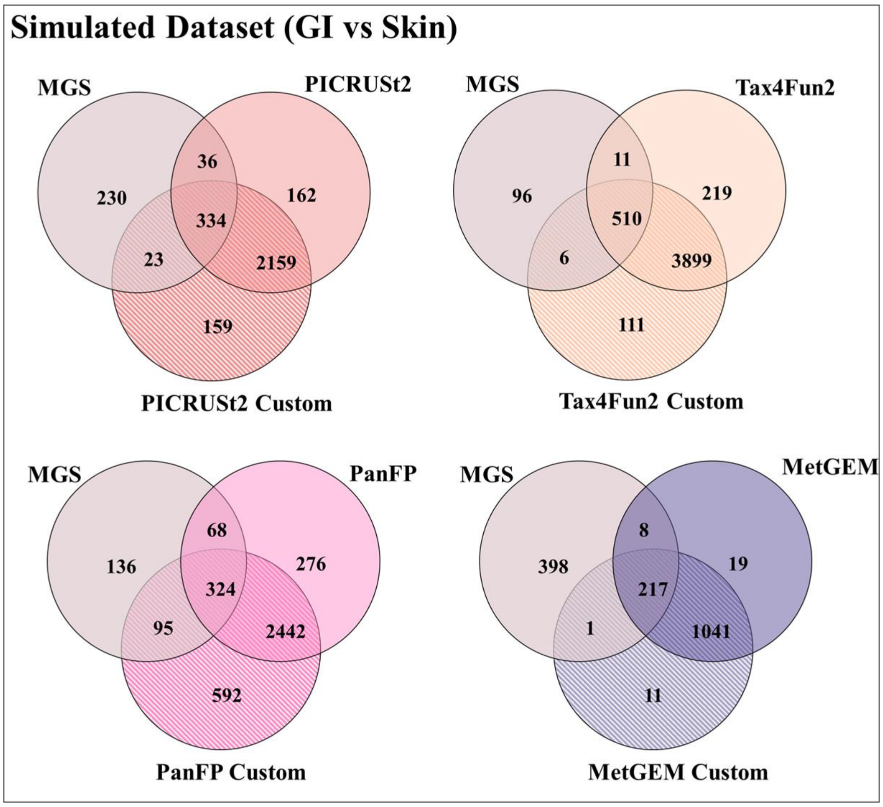

Figure S2: The overlap of significant KO terms between different functional inference tools and MGS results in the PopGen cohort. Overlapping significant KO (unadjusted p < 0.05) terms can be used as a quantitative indicator of the accuracy of the predictions.

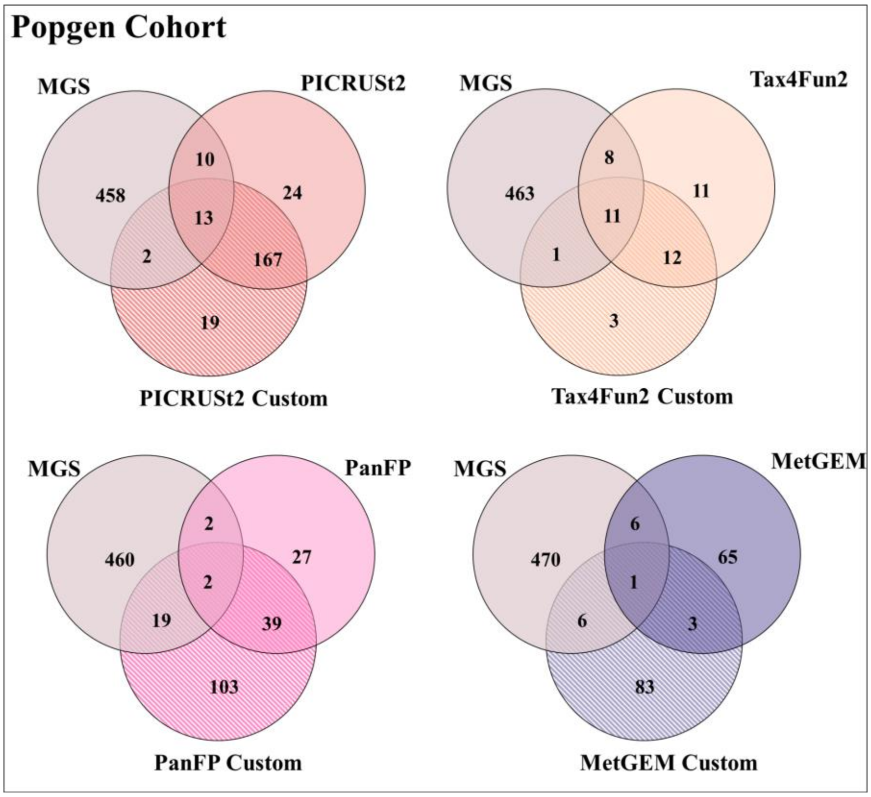

Figure S3: The overlap of significant KO terms between different functional inference tools and MGS results in the PopGen cohort. Overlapping significant (unadjusted p < 0.05) KO terms can be used as a quantitative indicator of the accuracy of the predictions.

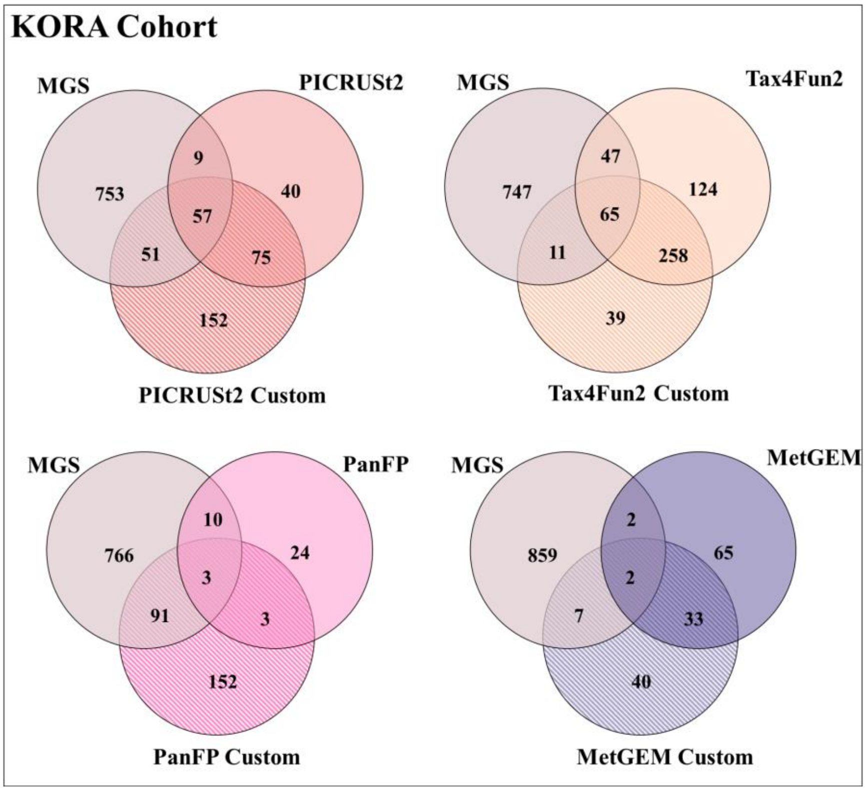

Figure S4: The overlap of significant KO terms between different functional inference tools and MGS results in the KORA cohort. Overlapping significant (unadjusted $p < 0.05$) KO terms can be used as a quantitative indicator of the accuracy of the predictions.

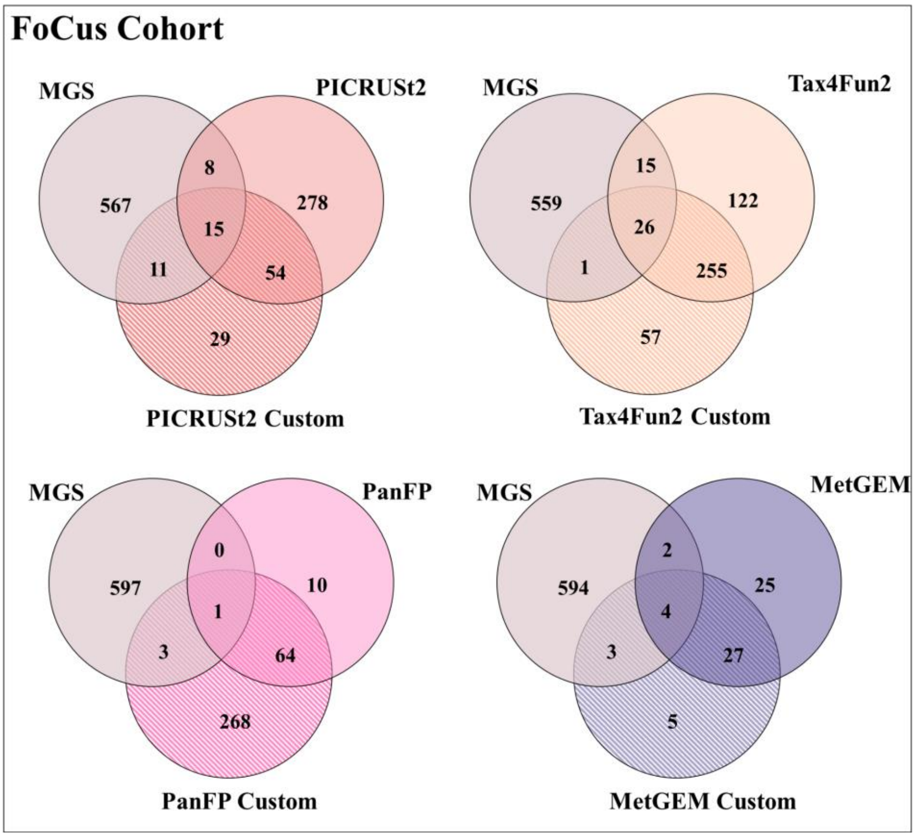

Figure S5: The overlap of significant KO terms between different functional inference tools and MGS results in the FoCus cohort. Overlapping significant (unadjusted $p < 0.05$) KO terms can be used as a quantitative indicator of the accuracy of the predictions.

The performance of functional inference tools varies widely across different KEGG functional categories

We also investigated if prediction performance varies across functional categories, suspecting that some categories may be easier to predict than others. In general, KO terms were organized into a hierarchical structure, with each term belonging to a KEGG higher-level category using categorize_by_function.py provided by PICRUSt2. For example, some categories may be more well-defined or have more established biological functions, making it easier to accurately predict the presence or abundance of related KO terms. Conversely, other categories may be more complex or poorly understood, leading to poorer prediction performance. To investigate whether prediction performance varies across functional categories, we compared the accuracy of predicted KO terms within each KEGG higher-level category. By doing so, we can identify which functional categories are particularly challenging to predict and whether there are any trends or patterns in the data that could explain these difficulties. This information could be useful for refining predictive models and improving the accuracy of predictions overall.

Level 2 KEGG functional categories further subdivide the level 1 categories into more specific functional categories. For example, the level 2 category "Carbohydrate metabolism" is a subset of the level 1 category "Metabolism." The KO terms themselves are organized into level 3 functional categories, which provide even more specific information about the functions of

individual genes or gene products. Hence, to evaluate the performance of functional inference tools at different levels of resolution, the predicted KO term abundances were aggregated at each level of the hierarchy using picrust1_categorize_by_func.R script. The categorization was based on a legacy table of mappings from KOs to the BRITE hierarchy, which was used as a reference. Once the KO terms were aggregated at the KEGG functional categories at each level, the Wilcoxon rank-sum test was performed to test for significant differences in KO abundances between the disease and healthy groups in each cohort.

We evaluated the results of inference tools considering significant changes observed in MGS data as ground truth using the F1, recall, and precision scores. Interestingly, we found that, except for the CRC cohort (S7), functional profiling tools did not identify any true positives, indicating that they did not identify any significant functional pathways related to the disease state in the other cohorts (KORA, PopGen, and FoCus). Cancer-associated dysbiosis might lead to more substantial changes in the metagenome than in other diseases, which could be better reflected in the 16S rRNA data. However, further data are needed to corroborate this.

PICRUSt2, PanFP, and Tax4Fun2 performed well at the level 3 functional categories. They rely on reference-based approaches to infer functional pathways in microbial communities, which may be beneficial for detecting pathways at level 3. MetGEM struggled at level 2 and level 3 functional categories. PanFP, which uses a pan genome-based approach, may be more effective at predicting functional pathways at level 2 because it does not rely on reference genomes or genome annotation data but instead uses a database of pangenomes from multiple species to predict metabolic pathways. This approach may be more flexible and better able to handle the diversity of microbial communities in different environments. Throughout our results, we consistently noted that there was no discernible positive effect when employing customized copy number normalization techniques.

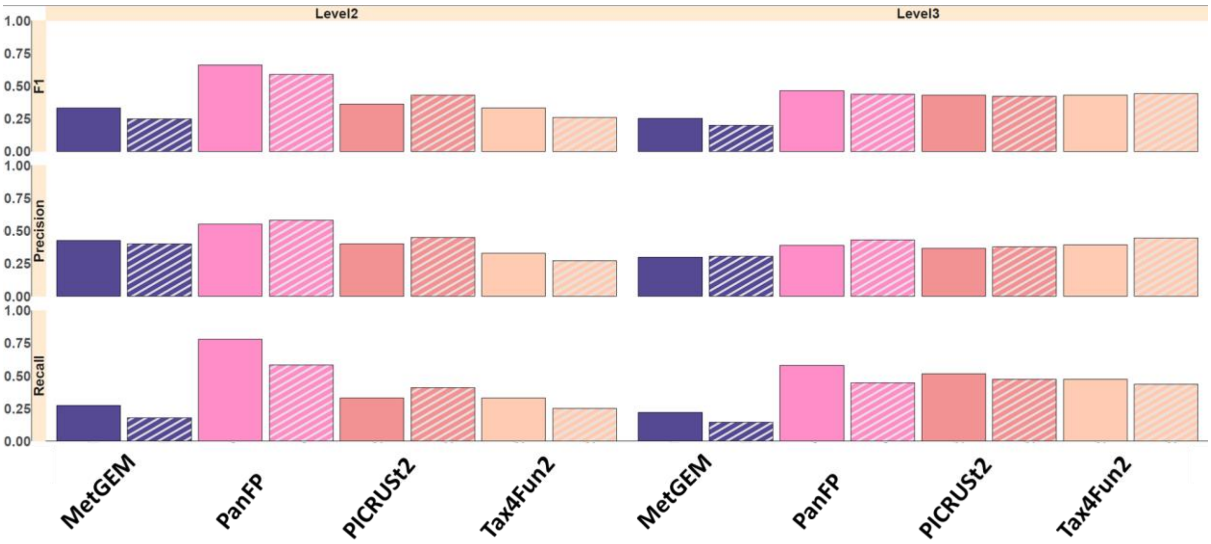

Figure S6: Comparison of F1 score, recall, and precision for predicting the occurrence of KEGG functional categories in CRC cohorts versus MGS data across three KEGG functional category levels.

As the next step, we selected specific functional categories related to each disease cohort and compared the differential abundance of individually detected KO terms within these categories across the tools. For instance, in the CRC cohort, we focused on glycan biosynthesis [2,3] and lipid metabolisms [4], which have been reported to be overrepresented in CRC patients.

We identified significant KO terms (unadjusted p-value < 0.05) based on MGS results and compared them with those from functional inference tools. Within the biosynthesis and metabolism of glycans category, PanFP exhibited the highest overlap with significant terms, followed by PICRUSt2 and Tax4Fun2 (default and customized normalization). As expected, no overlapping significant KO terms were found between MGS and MetGEM.

Additionally, we assessed tool performance using KEGG functional categories related to lipid metabolism. For the FoCus, PopGen, and KORA cohorts, we concentrated on KEGG functional categories, such as carbohydrate metabolism and amino acid metabolic pathways, which have been reported to be enriched in obesity [5,6]. Overall, looking at the individual KO terms in specific functional categories also proved that the functional inference tools cannot be considered to test the functional properties of microbes.

In summary, our analysis of individual KO terms within specific functional categories also indicates that functional inference tools may not be reliable for studying microbial functional properties.

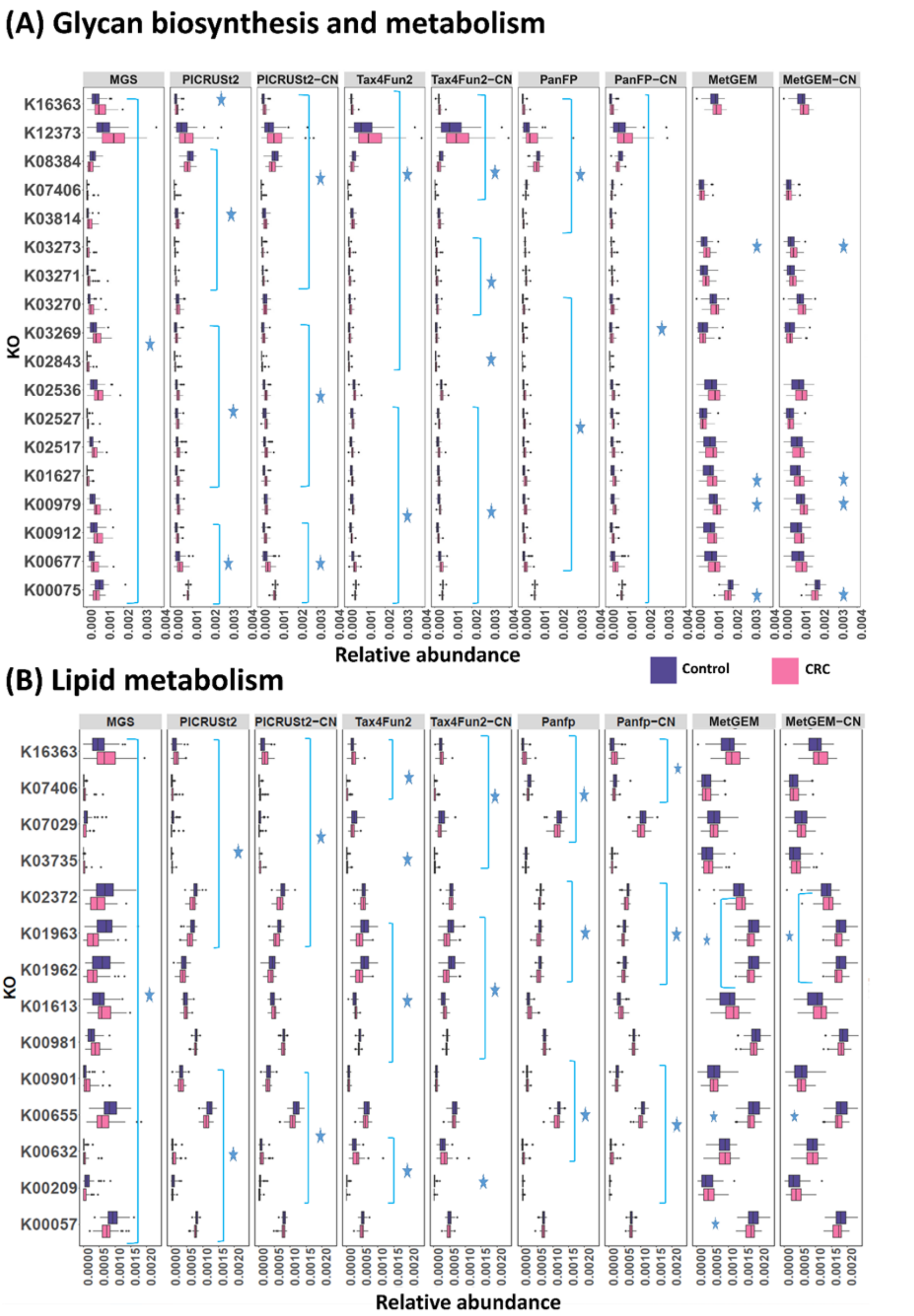
(A) Glycan biosynthesis and metabolism
MGS
PICRUSt2
PICRUSt2−CN
Tax4Fun2
Tax4Fun2−CN
PanFP
PanFP−CN
MetGEM
MetGEM−CN
K16363
K12373
K08384
K07406
K03814
K03273
K03271
K03270
K03269
K02843
K02536
K02527
K02517
K01627
K00979
K00912
K00677
K00075
KO
0.000
0.001
0.002
0.003
0.004
Relative abundance
Control
CRC
(B) Lipid metabolism
MGS
PICRUSt2
PICRUSt2−CN
Tax4Fun2
Tax4Fun2−CN
Panfp
Panfp−CN
MetGEM
MetGEM−CN
K16363
K07406
K07029
K03735
K02372
K01963
K01962
K01613
K00981
K00901
K00655
K00632
K00209
K00057
KO
0.0000
0.0005
0.0010
0.0015
0.0020
Relative abundance

Figure S7: Comparison of relative abundance distributions among major KEGG gene categories such as (A) glycan biosynthesis metabolism and (B) lipid metabolism between functional profiles produced by inference tools and those derived from MGS in the CRC cohort. A Wilcoxon rank-sum test was performed to compare the relative abundance between healthy and diabetes groups. The threshold of a p-value (unadjusted) of less than 0.05 indicated a significant difference (*).

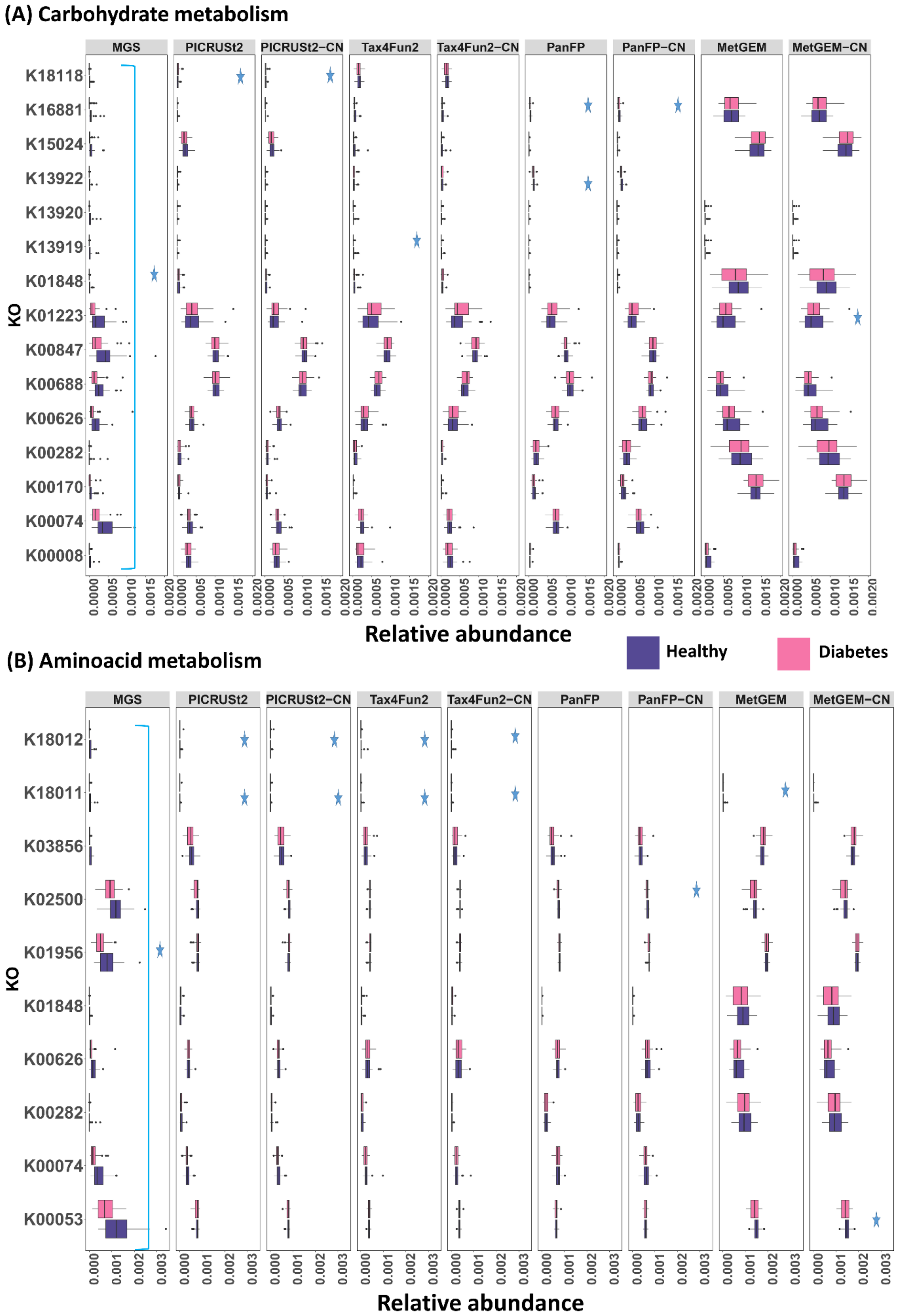
(A) Carbohydrate metabolism
MGS
PICRUSt2
PICRUSt2−CN
Tax4Fun2
Tax4Fun2−CN
PanFP
PanFP−CN
MetGEM
MetGEM−CN
K18118
K16881
K15024
K13922
K13920
K13919
K01848
K01223
K00847
K00688
K00626
K00282
K00170
K00074
K00008
KO
0.0000
0.0005
0.0010
0.0015
0.0020
Relative abundance
Healthy
Diabetes
(B) Aminoacid metabolism
MGS
PICRUSt2
PICRUSt2−CN
Tax4Fun2
Tax4Fun2−CN
PanFP
PanFP−CN
MetGEM
MetGEM−CN
K18012
K18011
K03856
K02500
K01956
K01848
K00626
K00282
K00074
K00053
KO
0.000
0.001
0.002
0.003
Relative abundance

Figure S8: Comparison of relative abundance distributions among major KEGG gene categories such as (A) carbohydrate metabolism and (B) amino acid metabolism between functional profiles produced by inference tools and those derived from MGS in the KORA cohort. A Wilcoxon rank-sum test was performed to compare the relative abundance between healthy and diabetes groups. A p-value (unadjusted) of less than 0.05 indicated a significant difference (*).

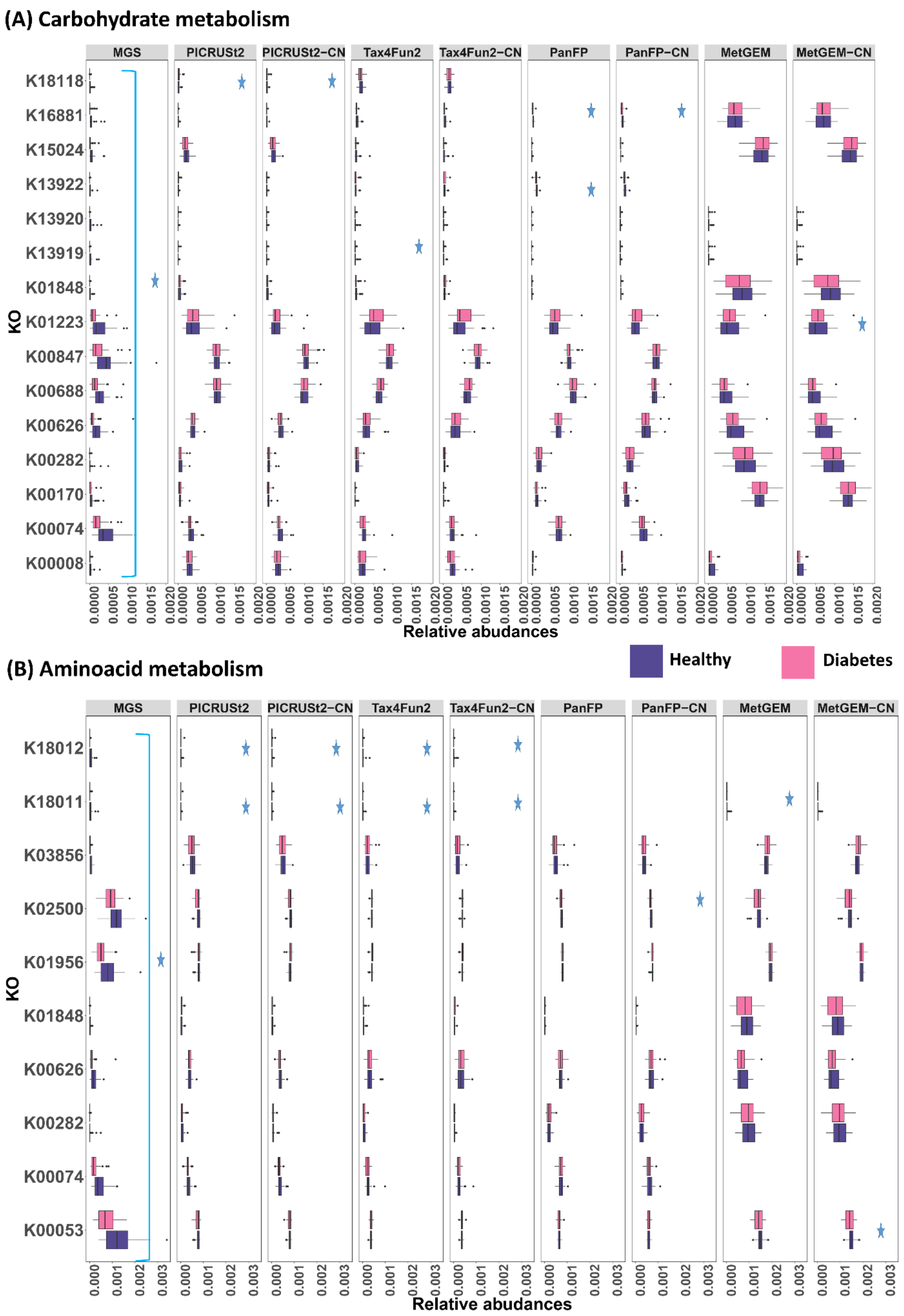
(A) Carbohydrate metabolism
MGS
PICRUSt2
PICRUSt2−CN
Tax4Fun2
Tax4Fun2−CN
PanFP
PanFP−CN
MetGEM
MetGEM−CN
K18118
K16881
K15024
K13922
K13920
K13919
K01848
K01223
K00847
K00688
K00626
K00282
K00170
K00074
K00008
KO
0.0000
0.0005
0.0010
0.0015
0.0020
Relative abudances
Healthy
Diabetes
(B) Aminoacid metabolism
MGS
PICRUSt2
PICRUSt2−CN
Tax4Fun2
Tax4Fun2−CN
PanFP
PanFP−CN
MetGEM
MetGEM−CN
K18012
K18011
K03856
K02500
K01956
K01848
K00626
K00282
K00074
K00053
KO
0.000
0.001
0.002
0.003
Relative abudances

Figure S9: Comparison of relative abundance distributions among major KEGG gene categories such as (a) carbohydrate metabolism and (b) amino acid metabolism between functional profiles produced by inference tools and those derived from MGS in the PopGen cohort. A Wilcoxon rank-sum test was performed to compare the relative abundance between healthy and obese groups. A p-value (unadjusted) of less than 0.05 indicated a significant difference (*).

(A) Carbohydrate metabolism

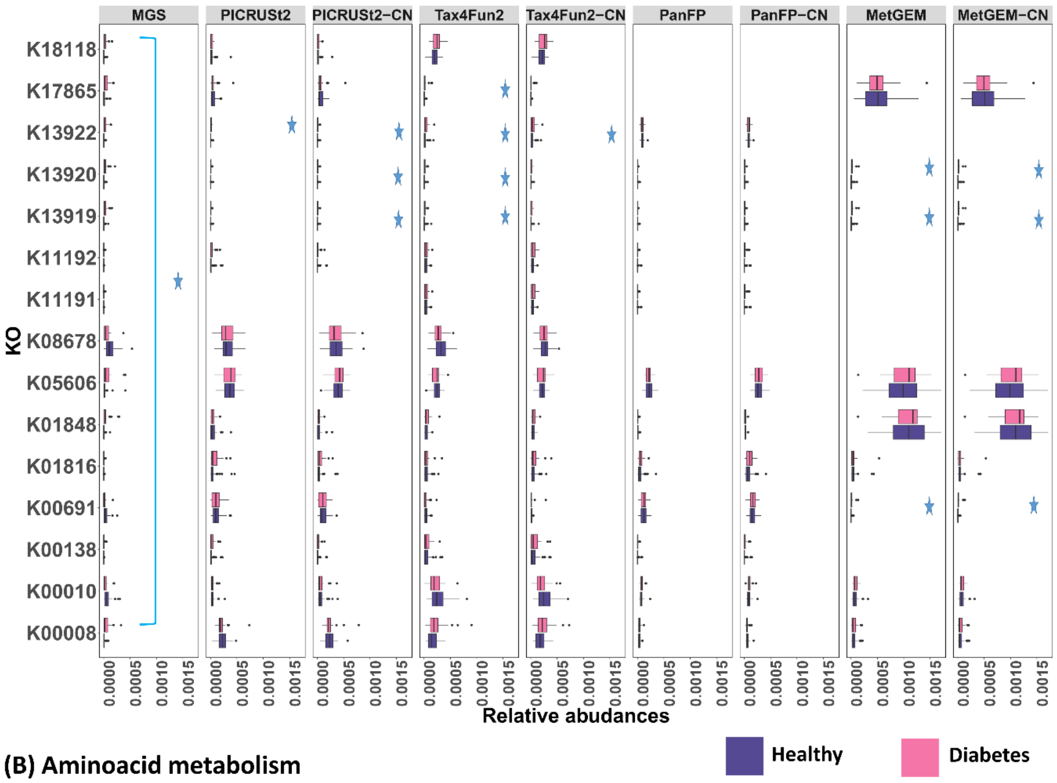

(B) Aminoacid metabolism

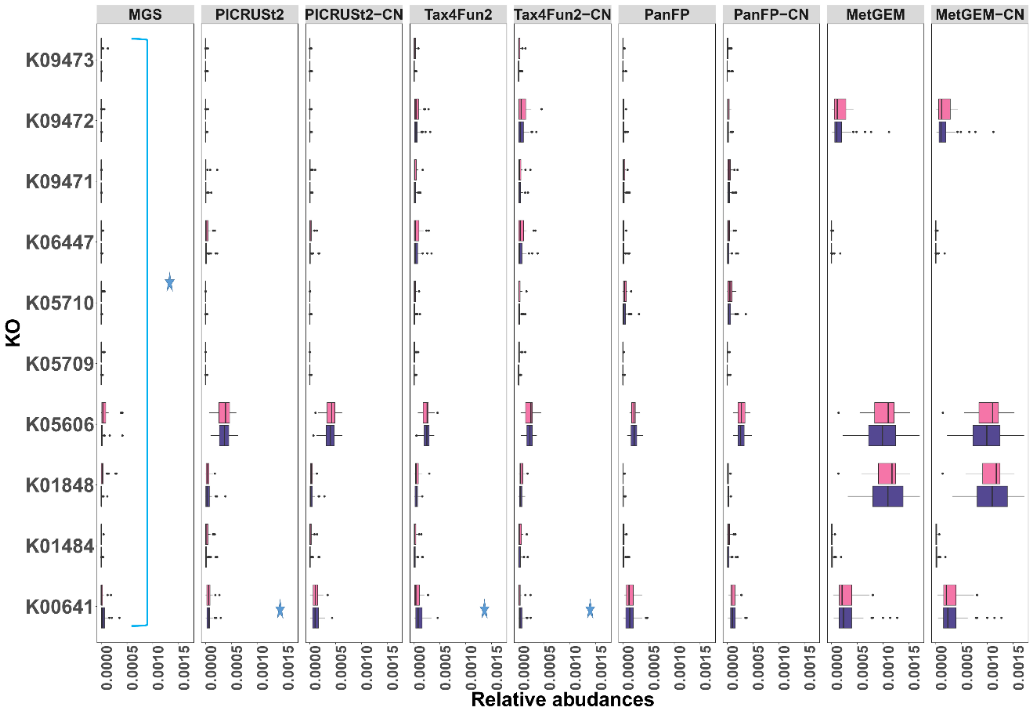

Figure S10: Comparison of relative abundance distributions among major KEGG gene categories such as (a) carbohydrate metabolism and (b) amino acid metabolism between functional profiles produced by inference tools and those derived from MGS in the FoCus cohort. A Wilcoxon rank-sum test was performed to compare the relative abundance between healthy and obese groups. A p-value (unadjusted) of less than 0.05 indicated a significant difference (*)

1. Sun S, Jones RB, Fodor AA. Inference-based accuracy of metagenome prediction tools varies across sample types and functional categories. Microbiome. 2020;8: 46.

2. Brockhausen I. Pathways of O-glycan biosynthesis in cancer cells. Biochim Biophys Acta. 1999;1473: 67–95.

3. Liu J, Huang X, Chen C, Wang Z, Huang Z, Qin M, et al. Identification of colorectal cancer progression-associated intestinal microbiome and predictive signature construction. J Transl Med. 2023;21: 373.

4. Zhou H, Chen Y, Xiao Y, Wu Q, Li H, Li Y, et al. Evaluation of the ability of fatty acid metabolism signature to predict response to neoadjuvant chemoradiotherapy and prognosis of patients with locally advanced rectal cancer. Front Immunol. 2022;13: 1050721.

5. Duan M, Wang Y, Zhang Q, Zou R, Guo M, Zheng H. Characteristics of gut microbiota in people with obesity. PLoS One. 2021;16: e0255446.

6. Bombin A, Yan S, Bombin S, Mosley JD, Ferguson JF. Obesity influences composition of salivary and fecal microbiota and impacts the interactions between bacterial taxa. Physiol Rep. 2022;10: e15254.
